# Escalating environmental inequalities in larger European regions: A data mining

**DOI:** 10.1016/j.dib.2023.109792

**Published:** 2023-11-10

**Authors:** Bardia Mashhoodi, Pablo Muñoz Unceta

**Affiliations:** a Landscape Architecture and Spatial Planning Group, Department of Environmental Sciences, Wageningen University & Research, the Netherlands, Address: P.O. box 47, 6700 AA Wageningen, the Netherlands; b Institute for Advance Architecture of Catalonia, Spain, Address: C. de Pujades, 102, 08005 Barcelona, Spain

**Keywords:** Gini, Environmental justice, Nuts regions, Europe, EU, Spatial inequality, Environmental inequality

## Abstract

Environmental inequality has been the focus of European scientists and policymakers in the past decades. This database is prepared to provide researchers with a multiscale, multivariate database on environmental inequality across different scales, i.e. the so-called NUTS region levels. To do so, the database offers the population-weighted average and Gini coefficient at four European NUTS region levels (NUTS 0, NUTS 1, NUTS 2, NUTS 3) over exposure to air pollution (NO2, O3, PM10, PM2.5), summer land surface temperature (LST), and Tree and Non-tree vegetated surfaces. The dataset can be used to compare and map the magnitude of inequalities related to each of the environmental hazards/services. Furthermore, it is helpful to identify the levels of scales with the highest and lowest levels of environmental inequality. To this end, this manuscript provides histograms and maps to present the potential for the use of the database.

Specifications Table

**Table:** 

Subject	Geographical Information System
Specific subject area	Environmental inequality
Data format	Raw and analysed
Type of data	GIS data (.shp; .tif); MS Excel (.xlsx); Image (.jpg)
Data collection	The database is prepared by downloading GIS data from different sources, spatially superimposing them using ArcGIS Pro version 2.9 “Sample” function, and using weighted Gini function in the “inequalipy” package in Python.
Data source location	Europe (54.5260° N, 15.2551° E)
Data accessibility	Repository name: Mendeley Data identification number (DOI): 10.17632/5czndsmdmp.2 Direct URL to data: https://data.mendeley.com/datasets/5czndsmdmp/2
Related research article	NA

## 1 Value of the Data

• The database offers the possibility of comparing different types of environmental inequalities - air pollution, land cover, and surface temperature - at different levels of scales, i.e. the so-called European NUTS0, NUTS1, NUTS2, NUTS3. In this respect, the database offers a novel possibility for multivariate, multiscale environmental inequality studies at the continental scale.
• The database enables human geographers, environmental scientists and regional studies experts to examine to what extent environmental inequalities are associated with geographical factors such as demography, urbanity and spatial distribution of urban-rural areas, climate types, and land cover. The database also allows for studying the associations between environmental inequalities and other kinds of inequality, namely income, gender and ethnic inequalities, across the European regions.
• The database can be further used for health-related studies and spatial planners to identify the regions with high exposure to hazards such as air pollution and surface temperature and those deprived in terms of access to green spaces. This can pave the way for further spatial interventions - e.g., land use or transport modification and provision of health services.

## 2 Data Description

### 2.1 Data analysis

The database comprises measures of environmental inequality in exposure to air pollution and land surface temperature in the EU regions. Environmental inequality is measured using the population-weighted gini coefficient. To calculate the population-weighted Gini coefficient in the region *g* ∈ [1,G], the 1 km x 1 km raster data, hereinafter called *cells*, on population and environmental hazards/services (air pollution, land surface temperature, presence of trees, or presence of non-tree vegetated surfaces) are used. In the first step, the cells are ranked based on their population, with r ∈ [1,R] showing the rank of a cell. *P_gr_* represents the population of cell rank *r* in region *g*, and *W_gr_* is the weight of cell rank r (see Eq. (1)).

(1) $$W_{gr}=P_{gr}/\sum_{1}^{R}P_{gr}$$

Subsequently, cell weights are used to calculate the population-weighted average of environmental hazard/service exposure in region *g* (see Eq. (2)):

(2) $$A_{g}=\sum_{1}^{R}W_{gr}H_{gr}$$

where $H_{gr}$ shows the magnitude of hazard in the cell ranked *r* in region *g*. (Note that the ranks are based on cell population.) Adapted from Lerman and Yitzhaki [1], the population-weighted Gini coefficient, i.e. the measurmenet of hazard-exposure inequality, is calculated as follows (Eq. (3)):

(3) $$G_{g}=2\sum_{1}^{R}W_{gr}\left(H_{gr}-A_{g}\right)\left({\hat{F}}_{gr}-\bar{F}\right)/A_{g}$$

where *G_g_* represents the population-weighted Gini coefficient of the environmental factor in grid *g*, ${\hat{F}}_{gr}$ showing the weighted cumulative distribution of the environmental factor (Eq. (4)), and $\bar{F}\;$representing the average of ${\hat{F}}_{gr}$:

(4) $${\hat{F}}_{gr}=\sum_{i=1}^{r-1}W_{gi}+W_{gr}/2$$

### 2.2 Raw data

**Fig. 1:**
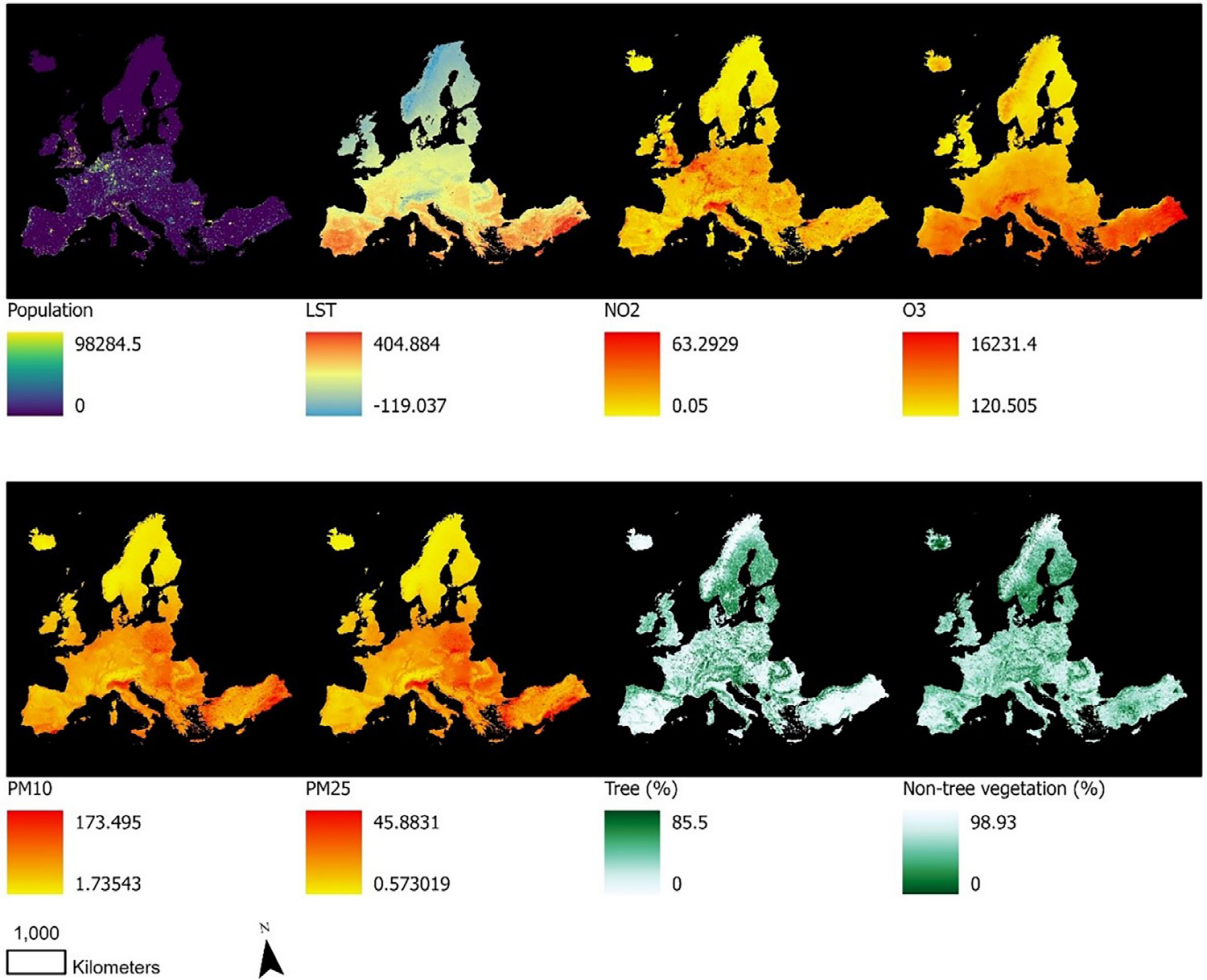
The data on population and environmental hazard/services.

The raw data (saved in Data_DiB_EJ_NUTS\ GIS_DiB_EJ.gdb) includes the 1 x 1 km raster files of population and environmental hazard/services (Fig. 1):

• The raster files (.tif) on population at 1 km x 1 km scale, as estimated by the world pop (WorldPop2019.tif) [2];
• The raster files on air pollution including No2 (no2_avg19_.tif) [3], O3 (o3_s35_19.tif) [4], PM10 (pm10_avg19_.tif) [5], PM2.5 (pm25_avg19_.tif) [6];
• The raster file on average LST in summer (21 June to 23 September) 2019 (LST_Summer2019_DayNight_cel.tif) [7]. Due to the sheer size of the raw data, only the aggregated data for Europe is stored in the database.
• The raster files on the percentage of vegetated tree (Tree_1 km.tif) and vegetated non-tree (NTree_1 km.tif) land covers [8].

**Fig. 2:**
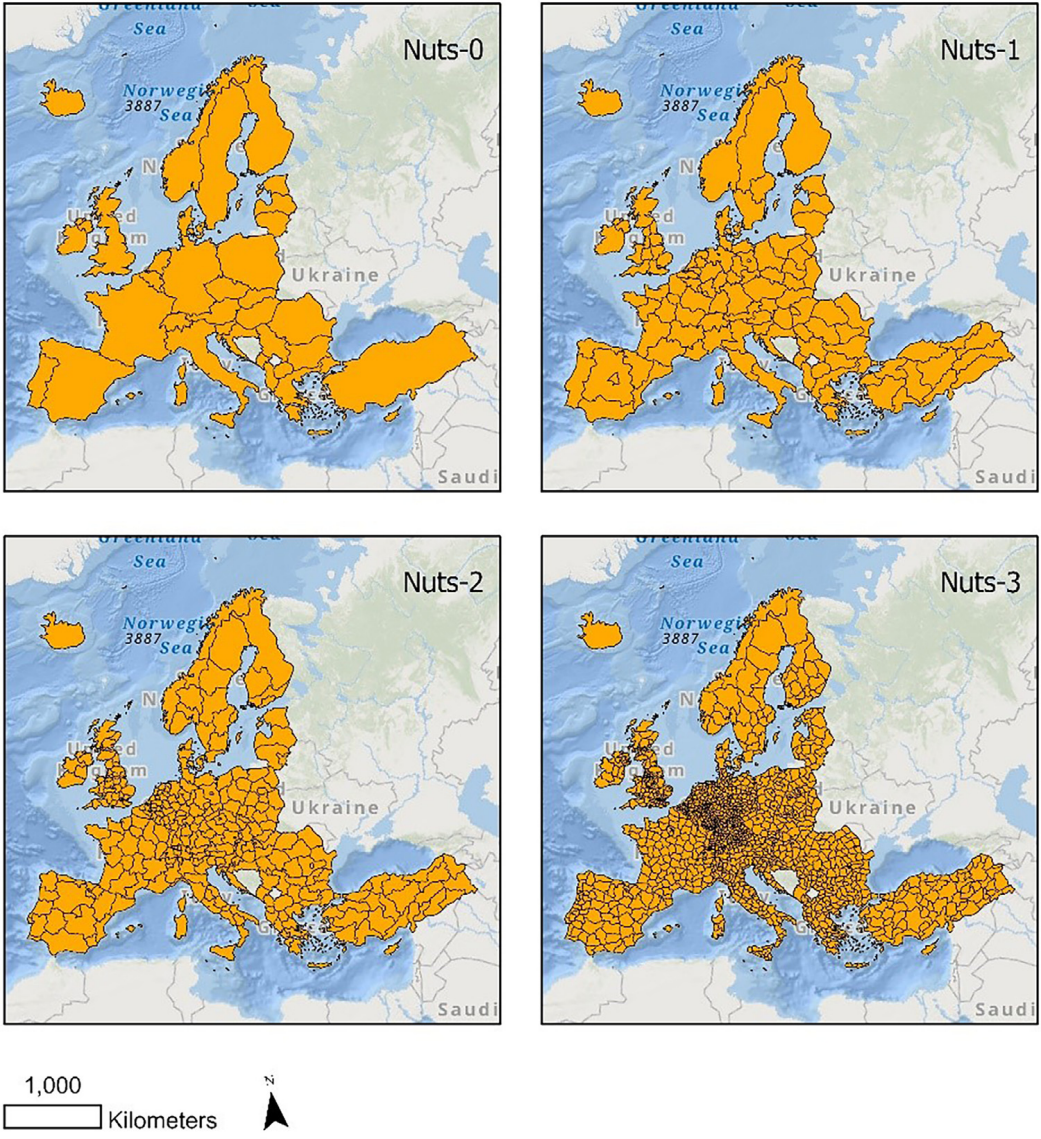
Inequality analyses are conducted in four scales, i.e. the so-called NUTS region levels.

The population-weighted Inequalities of each environmental hazrad/service are calculated in four scales, the so-called NUTS regions scales (Fig. 2). The GIS file with the boundaries of regions (.shp) is saved as *NUTS_RG_20M_2021_3035.shp* [9].

### 2.3 Population-weighted Gini and average of environmental hazards/services in the NUTS regions

The final database (Data_DiB_EJ_NUTS\ Gini_Weighted Average.xlsx) includes the population-weighted Gini and population-weighted average of the environmental hazards and services. Table 1 shows the fields in the database and their definitions.

**Table 1:** Description of the fields in the file on population-weighted Gini and population-weighted average of exposure to environmental hazards/services.

Variable acronym	Description
NUTS_ID	NUTS code corresponding to the raw data on NUTS boundaries
NUTS_Level	Indicating the level of NUTS (0 to 3)
GINI_LST	Population-weighted Gini coefficient of LST
WA_LST	Population-weighted average of LST
GINI_NO2	Population-weighted Gini coefficient of NO_2_
WA_NO2	Population-weighted average of NO_2_
GINI_O3	Population-weighted Gini coefficient of O_3_
WA_O3	Population-weighted average of O_3_
GINI_PM10	Population-weighted Gini coefficient of PM_10_
WA_PM10	Population-weighted average of PM_10_
GINI_PM25	Population-weighted Gini coefficient of PM_2.5_
WA_PM25	Population-weighted average of PM_2.5_
GINI_TREE	Population-weighted Gini coefficient of surfaces covered by trees
WA_TREE	Population-weighted average of surfaces covered by trees
GINI_NTREE	Population-weighted Gini coefficient of surfaces covered by non-tree vegetation
WA_NTREE	Population-weighted average of surfaces covered by non-tree vegetation

**Fig. 3:**
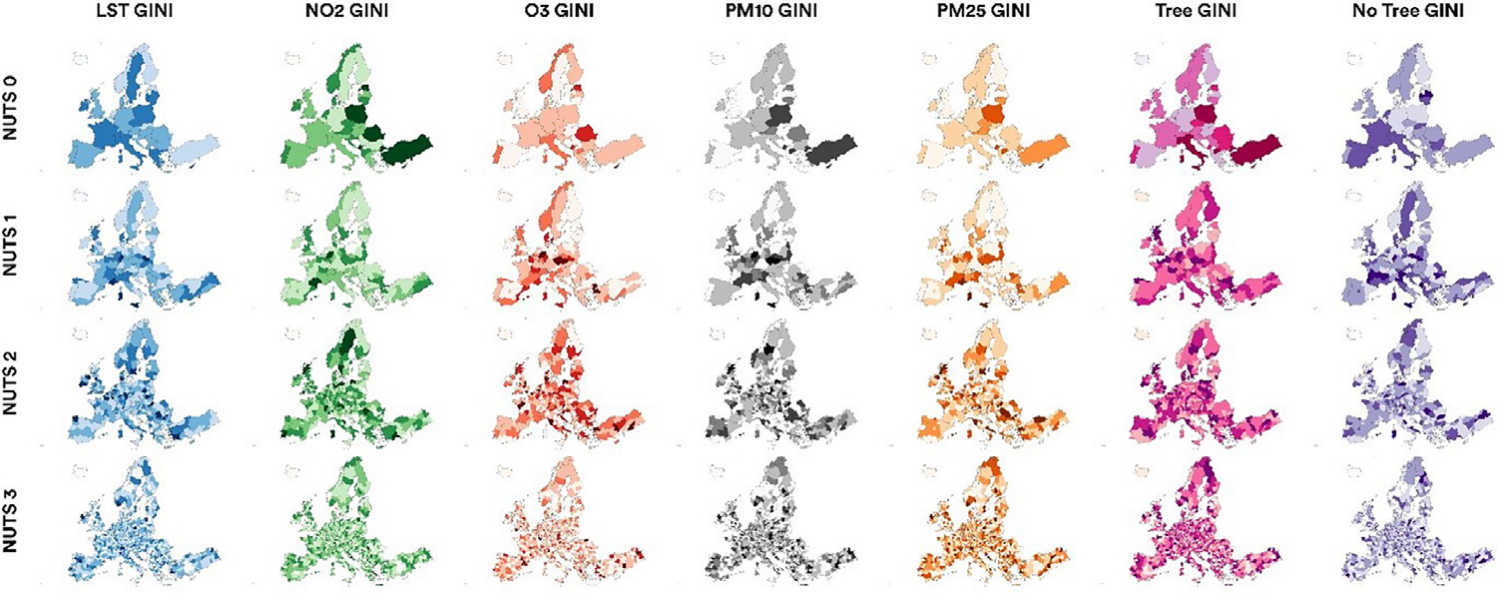
Population-weighted Gini inequality in NUTS regions.

Using NUTS codes, the database on inequality and average exposure can be joined to the NUTS shape file (see Section 1.1) and be used for mapping inequalities across different regional levels (Fig. 3). The high-resolution inequality map is provided in the database (Data_DiB_EJ_NUTS\ Comparative_GINI map.jpg).

**Fig. 4:**
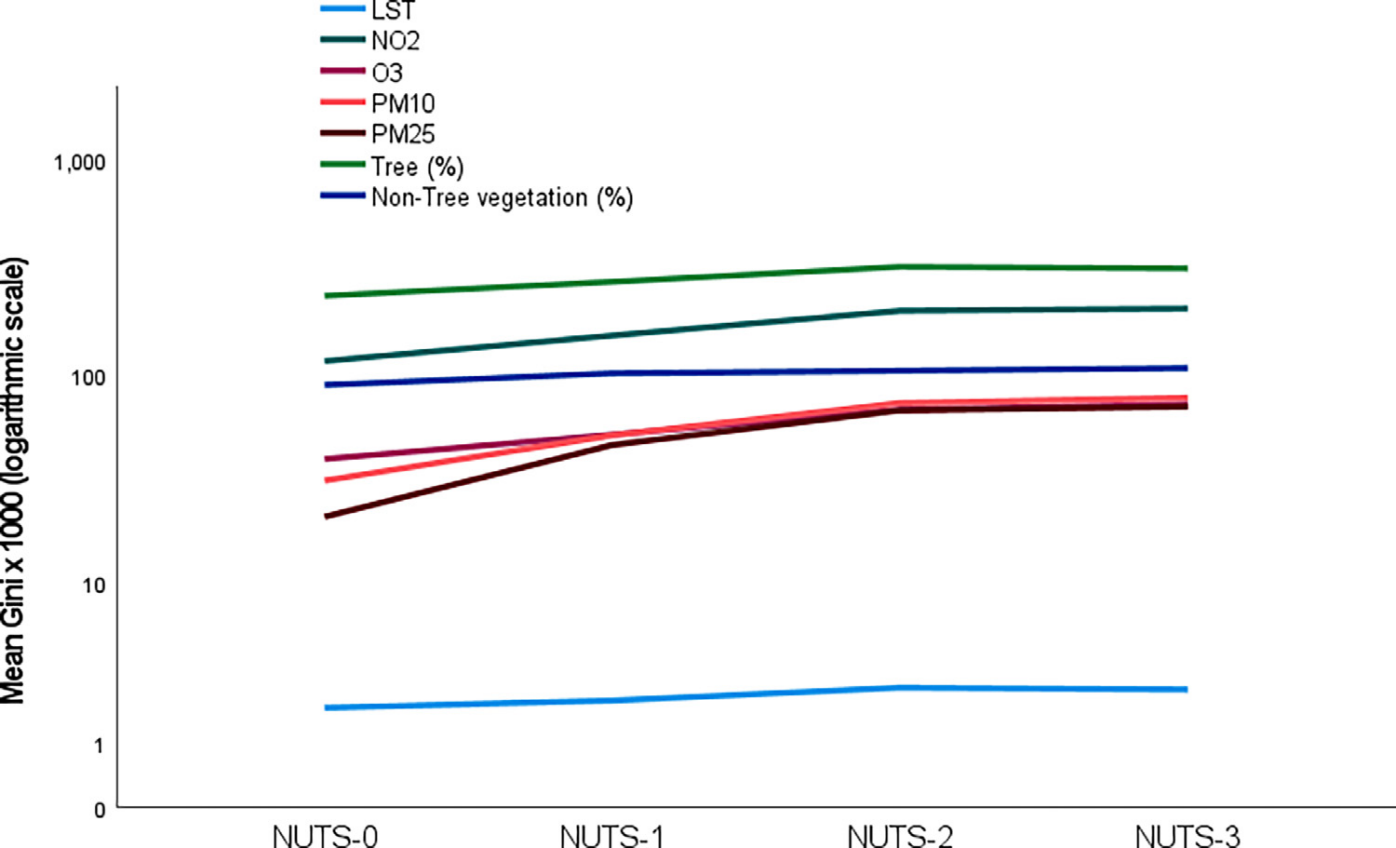
Comparison of inequalities of the environmental hazards/services at different regional levels.

Furthermore, the dataset can be used to compare the inequalities of different environmental factors (Fig. 4).

**Fig. 5:**
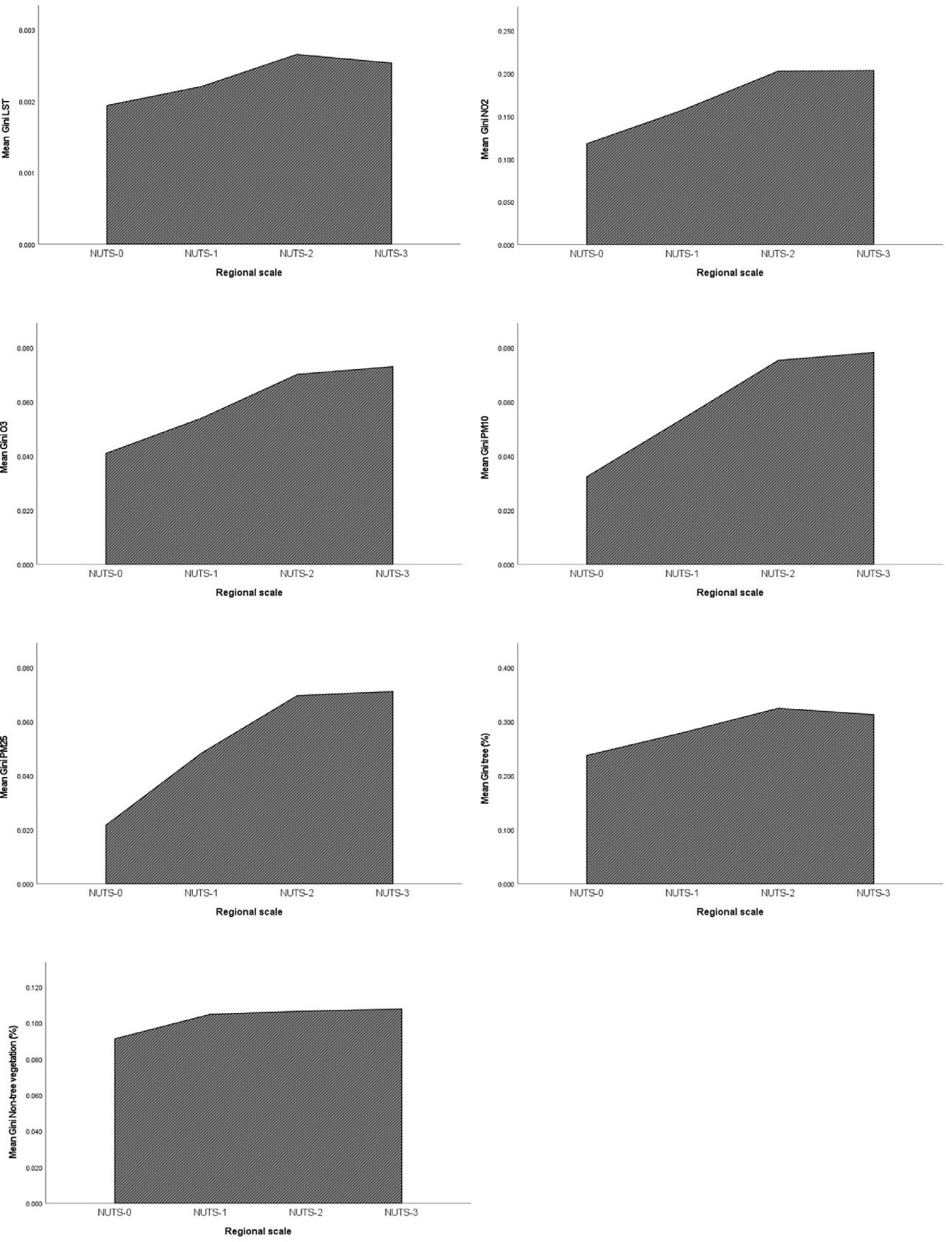
Variations of environmental factors’ inequalities across the regional scales.

In detail, the database provides insights on variatiosn of inequality across the levels of scale (Fig. 5).

## 3 Experimental Design, Materials and Methods

The workflow to generate the database is as follows. The raw raster data are downloaded and collected from different sources. The resolution of all the raster files is set at 1 km x 1 km, and pixels are "snapped" at the population raster to have the exact spatial match. When the raster values needed to be aggregated from 250 m x 250 m to 1 km to 1 km, the mean value of the pixels is used. To do so, the "resample" function of ArcGIS Pro 2.9 is used. Subsequently, the "sample" function in ArcGIS pro produces a data file (.dbf) with the values of all overlapping pixels. Each row of the data file includes (x,y) coordinates of the centroid of the pixel in question, the identification codes of the European regions the pixel is located in, and the population and environmental hazards/services values. The data file is imported in Python and, using the weighted Gini function of the "inequalipy", the population-weighted Gini coefficient and the population-weighted average of the environmental hazards/services are obtained.

The database can be used to study the impact of unequal exposure to heat and different landcover types on energy consumption (similar to [10,11,12]), energy poverty (similar to [13,14]), environmental inequality among socioeconomic groups (similar to [15,16]), and distribution of electric vehicles to reduce air pollution inequality (by approaches similar to [17,18,19]).

## Limitations

Not applicable.

## Ethics Statement

The authors have read and follow the ethical requirements for publication in Data in Brief and confirming that the current work does not involve human subjects, animal experiments, or any data collected from social media platforms.

## CRediT authorship contribution statement

**Bardia Mashhoodi:** Conceptualization, Methodology, Software, Data curation, Writing – original draft, Visualization, Investigation, Validation, Writing – review & editing. **Pablo Muñoz Unceta:** Conceptualization, Methodology, Software, Data curation, Writing – original draft, Visualization, Investigation, Validation, Writing – review & editing.

## Data Availability

Environmental inequality in European NUTS regions (Original data) (Mendeley Data)
